# Analysis of an Ultra-Wideband, Perfectly Absorptive Fractal Absorber with a Central Square Nanopillar in a Cylindrical Structure with a Square Hollow

**DOI:** 10.3390/ma16216898

**Published:** 2023-10-27

**Authors:** Shang-Te Tsai, Jo-Ling Huang, Pei-Xiu Ke, Cheng-Fu Yang, Hung-Cheng Chen

**Affiliations:** 1 Business School, Yulin Normal University, Yulin 537000, China; feilongzz@gmail.com; 2 Department of Chemical and Materials Engineering, National University of Kaohsiung, Kaohsiung 811, Taiwan; a1103132@mail.nuk.edu.tw (J.-L.H.); m1115615@mail.nuk.edu.tw (P.-X.K.); 3 Department of Aeronautical Engineering, Chaoyang University of Technology, Taichung 413, Taiwan; 4 Prospective Technology of Electrical Engineering and Computer Science, National Chin-Yi University of Technology, Taichung 411, Taiwan

**Keywords:** ultra-wideband, fractal absorber, perfect absorptivity, square pillar, hollow cylindrical structure

## Abstract

In this study, a fractal absorber was designed to enhance light absorptivity and improve the efficiency of converting solar energy into electricity for a range of solar energy technologies. The absorber consisted of multiple layers arranged from bottom to top, and the bottom layer was made of Ti metal, followed by a thin layer of MgF_2_ atop it. Above the two layers, a structure comprising square pillars formed by three layers of Ti/MgF_2_/Ti was formed. This pillar was encompassed by a square hollow with cylindrical structures made of Ti material on the exterior. The software utilized for this study was COMSOL Multiphysics^®^ (version 6.0). This study contains an absorption spectrum analysis of the various components of the designed absorber system, confirming the notion that achieving ultra-wideband and perfect absorption resulted from the combination of the various components. A comprehensive analysis was also conducted on the width of the central square pillar, and the analysis results demonstrate the presence of several remarkable optical phenomena within the investigated structure, including propagating surface plasmon resonance, localized surface plasmon resonance, Fabry–Perot cavity resonance, and symmetric coupling plasma modes. The optimal model determined through this software demonstrated that broadband absorption in the range of 276 to 2668 nm, which was in the range of UV-B to near-infrared, exceeded 90.0%. The average absorption rate in the range of 276~2668 nm reached 0.965, with the highest achieving a perfect absorptivity of 99.9%. A comparison between absorption with and without outer cylindrical structures revealed that the resonance effects significantly enhanced absorption efficiency, as evidenced by a comparison of electric field distributions.

## 1. Introduction

Optical metamaterials have captured significant attention within both the engineering and scientific communities. This heightened interest stems from their remarkable electromagnetic (EM) response, which has paved the way for a multitude of technological applications [1,2,3]. These specially crafted materials offer a versatile means of controlling the polarization, phase, and amplitude of incident radiation, all achieved at an intricate subwavelength scale [4]. Metamaterials are intricate structures primarily composed of nano-scale scatterers and resonators. These components are further complemented by meta-molecules, each characterized by a unique combination of arrangement, orientation, geometry, shape, and size. This intricate composition empowers them with the ability to finely manipulate electromagnetic interactions. In this framework, metasurfaces based on a negative refractive index (RI) have ushered in a new era of captivating applications. These applications include superlensing [5], where the resolution surpasses the limitations of traditional lenses; planar filters that can selectively pass or block certain wavelengths [6]; and optical cloaking [7], enabling objects to be concealed from specific wavelengths of light. Furthermore, metasurfaces find utility in wavefront manipulation [8], crafting tailored optical chirality [9], enhancing medical imaging techniques [10], and achieving a near-perfect absorption of incident radiation [11,12,13].

In the past, extensive research efforts were dedicated to developing optical absorbers that possessed both ultra-wideband properties and high absorptivity across various regions of the electromagnetic spectrum. The incorporation of metals and oxides in the design of absorbers with a high absorptivity is grounded in their inherent optical properties. Metals often display pronounced absorptivity within visible and near-infrared realms due to the excitation of surface plasmon resonances, facilitating the efficient conversion of energy into heat. This trait positions them as promising contenders for achieving elevated absorptivity across a broad spectrum of wavelengths. By contrast, oxides possess adaptable optical properties that can be finely tailored through adjustments to their composition and structure. This malleability allows for the precise regulation of absorption characteristics within specific wavelength ranges. Notably, oxide materials exhibit varying absorption degrees across diverse wavelengths, rendering them invaluable for the crafting of absorbers directed at distinct spectral domains. By carefully selecting and combining these materials, researchers can design absorbers that are skilled at capturing and converting incoming electromagnetic radiation into thermal energy. This achievement allows for the achieving of a high level desired for absorptivity performance. This approach is important across a wide range of applications, including harnessing solar energy and managing thermal systems. Numerous metals and oxides have been employed in the pursuit of designing materials with ultra-wideband and perfect absorptivity characteristics. Examples include metals such as Al, Cu, Mo, Ti, and various others, as well as oxides like TiO_2_, SiO_2_, MgF_2_, and several additional options [11,13,14].

The utilization of optical absorbers holds considerable importance within the broader realm of solar energy harvesting. This importance stems from their pivotal role in converting sunlight into practical forms of energy, typically manifesting as electricity or heat. Optical absorbers serve as indispensable components in a variety of solar energy technologies, including photovoltaic cells and solar thermal systems. Here is why their significance is noteworthy:

(a) Energy conversion: In photovoltaic cells, optical absorbers efficiently capture photons, instigating the photovoltaic effect and generating an electrical current. In solar thermal systems, these absorbers harness sunlight to heat a working fluid, which can be subsequently employed to generate electricity or supply heat.
(b) Enhanced efficiency: The efficacy of a solar energy system heavily relies on the absorber’s capability to proficiently capture and convert sunlight. A high-quality optical absorber can efficiency amplify energy conversion, culminating in a more productive solar energy system.
(c) Cost efficiency: Elevating the performance of optical absorbers can render solar energy technologies more economically viable by diminishing the overall cost per unit of energy generated. Improved efficiency equates to greater energy production, using the same quantity of materials and installation expenses.

The significance of employing optical absorbers in the realm of solar energy harvesting is rooted in their capacity to adeptly capture and convert sunlight into usable energy. This technology assumes a pivotal role in propelling the adoption of solar energy, diminishing our reliance on fossil fuels, and thereby contributing to a more sustainable and environmentally friendly energy landscape.

A fractal optical absorber is a specialized device meticulously engineered to enhance light absorption over a broad spectrum of wavelengths. These absorbers hold significant relevance in various practical applications, especially in domains where effective light absorption plays a pivotal role. Key areas where fractal optical absorbers find application encompass solar energy conversion, photodetectors, sensors, energy harvesting, and light emission control. The viability of employing fractal optical absorbers in practical scenarios hinges on a combination of factors, such as material properties, cost considerations, size constraints, and the specific demands of the application at hand. As research and technology continue to progress, the feasibility and utility of fractal optical absorbers across a diverse array of applications are expected to improve.

In previous times, prevailing methodologies for crafting optical absorbers hinged upon several pivotal technologies, and these encompassed different physical resonances or light traveling. In the propagation of surface plasmon, the plasmon travels in a reciprocating manner between the structure’s opposing ends; it is necessary for at least one dimension of the structure to be in proximity to or exceed the wavelength of the incident light. Moreover, the resultant waves generated through the propagation of these surface plasmon can be finely adjusted by manipulating the geometric characteristics of the metallic nanostructure [13]; the localized surface plasmon resonance and both the material’s thickness and the area of the nanoparticles’ surface play important roles. For example, changing the aspect ratio of a nanorod or a nanopillar (the ratio of its length to its diameter) can lead to shifts in the localized surface plasmon resonance peak wavelength [13,15]; By strategically implementing an anti-reflection layer with precisely selected characteristics, this gradual transition mitigates the abrupt refractive index shift that typically gives rise to substantial reflection. Consequently, incident light undergoes a seamless traversal across the anti-reflection layer and seamlessly enters the absorber, thereby resulting in heightened absorptivity [13,15]. The emergence of resonance within a metal–insulator–metal Fabry–Perot cavity acts as the fundamental catalyst for enhancing the absorptivity of an optical absorber. This resonant occurrence plays a pivotal role in concentrating and entrapping incident light within the confines of the cavity’s structure, consequently resulting in a noteworthy escalation of absorption efficiency [11,13,15]. 

Beyond this repertoire of techniques, an array of alternative approaches also exists, all contributing to the creation of optical absorbers distinguished by their exceptional attributes of ultra-wideband functionality and exceptional absorbency exceeding the 90% threshold. These novel methodologies, often grounded in cutting-edge research, propel the field forward by expanding the horizons of what is achievable. As researchers delve into innovative configurations, intricate designs, and new materials, the goal of attaining ultra-efficient optical absorbers becomes increasingly attainable. Designing an optical absorber using a fractal structure offers a multitude of advantages, rendering it an appealing approach for diverse applications [16,17]. Some of the key benefits encompass the following:

(a) The inherent self-similarity of fractals allows them to resonate at various frequencies, enabling them to effectively absorb a broad spectrum of light.
(b) By adjusting parameters within the fractal geometry, one can precisely tailor absorption properties to target specific wavelength ranges.
(c) The complex composition of fractal structures promotes increased interactions between light and matter, thereby enhancing the efficiency of light absorption.
(d) Fractal absorbers skillfully trap and confine light within their structures, which is a valuable characteristic with significant applications in fields like solar cells and sensors.

Moreover, fractal designs can be meticulously crafted to mitigate reflectance by harnessing the numerous internal reflections inherent in a fractal geometry. Such control over reflectance is of paramount significance in applications where mitigating light loss due to reflection is a pivotal concern.

However, it is crucial to emphasize that, while the fractal structures indeed present a plethora of benefits for designing highly efficient absorbers, their creation can entail intricate complexities. Employing fractal structures to realize absorbers boasting ultra-wideband capabilities and impeccable absorptivity demands meticulous attention to factors such as geometry, material properties, and simulation methodologies. Symmetric plasma modes refer to modes characterized by one or more axes of symmetry [18,19]. Within a plasma system exhibiting a certain level of symmetry, specific oscillatory or vibrational patterns exist that possess symmetry in certain directions while not necessarily maintaining symmetry in others. In certain plasma systems, this symmetry is present, implying that properties and behaviors in certain directions are identical or similar. When such symmetry is applied to plasma oscillations, it gives rise to symmetric plasma modes. Symmetric plasma modes offer valuable advantages in terms of simplified analysis, enhanced stability, diagnostic insights, efficient energy transfer, mode selectivity, and design guidance. Their presence and manipulation contribute to advancing the understanding of plasma behavior and optimizing the performance of plasma-based applications. 

In this research endeavor, we embarked upon the design, analysis, and optimization of a novel fractal absorber. The absorber’s intricate architecture featured a central square pillar enveloped by a cylindrical structure, enclosed in its entirety by an outer square hollow. The reason for us using a square pillar within a square cavity of a cylinder is hoped to primarily generate symmetrical coupling plasma modes. Symmetrical coupling plasma modes refer to electromagnetic field modes with symmetric characteristics that can be confined to the conductor walls of the cylinder and can be generated within the plasma inside the cylinder. The presence of the square pillar has an impact on these symmetrical coupling plasma modes. It can be used to control the propagation and interaction of these modes. The geometric shape of the square pillar guides the electromagnetic field, causing it to reflect, refract, and interfere in the space surrounding the pillar. This results in the coupling and interaction of plasma modes between the cylinder and the square pillar. In other words, the principle of using a square pillar within a square cavity of a cylinder to achieve symmetrical coupling plasma modes is based on the interaction of electromagnetic fields in this unique geometric structure. In the structure under investigation, the central square pillar is situated at the center of a cylindrical structure with a square hollow. Therefore, it is evident that the width of the central square pillar will affect its symmetrical coupling with the plasma modes of the cylindrical structure.

Our primary objective was to explore the potential for achieving exceptional performance in terms of ultra-wideband absorption across the spectrum of ultraviolet-B (UV-B), visible light, and near-infrared-B (IR-B). The heart of our innovation lay in the incorporation of an advanced three-layered square pillar fractal structure. This intricate arrangement is meticulously crafted to enhance the absorber’s capabilities, and our endeavor is centered around showcasing a multitude of extraordinary optical phenomena that has manifested within this intricate structure. Among these phenomena are the captivating propagating surface plasmon resonance, the intriguing localized surface plasmon resonance, the resonant behavior of the Fabry–Perot cavity, and the symmetrical coupling plasma modes. The synergistic interplay of these phenomena culminates in the realization of truly exceptional absorption characteristics within our meticulously designed absorber. In particular, our achievements are noteworthy. We managed to attain an impressively broad ultra-wideband absorption range spanning from 276 to 2668 nm, and notably, the efficiency of this absorption exceeded the 90.0% threshold. Moreover, this absorptive prowess remained consistently remarkable throughout the entire wavelength range, boasting an average absorptivity of 0.965. This substantiates the absorber’s capability to consistently capture and harness electromagnetic energy across a diverse spectrum of wavelengths.

## 2. Simulation Process

**Figure 1:**
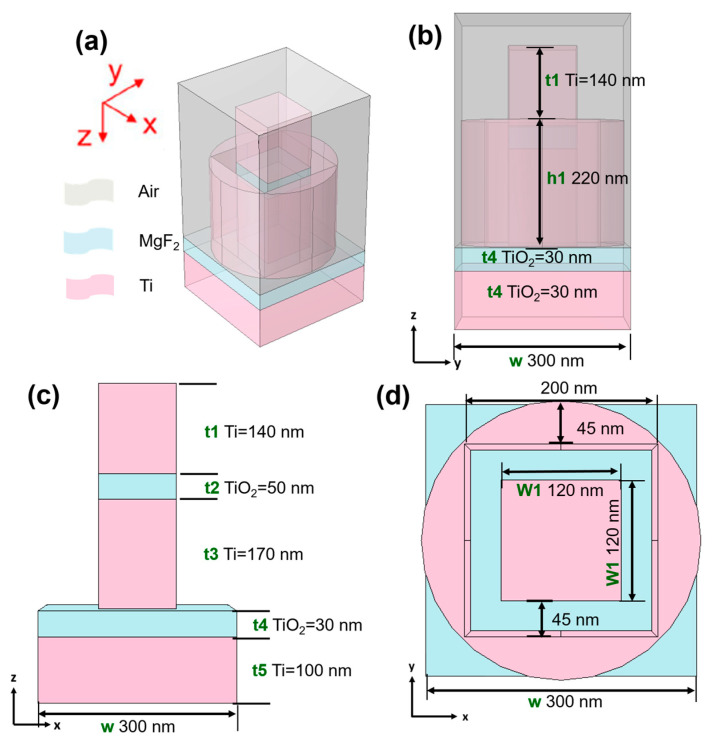

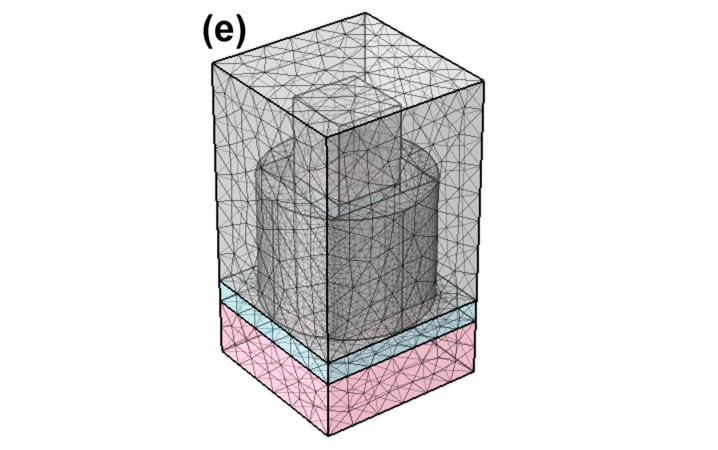
(**a**) Perspective view, (**b**) side view, and (**c**) top view of the designed absorber, (**d**) the side view of the designed central square pillar, and (**e**) the mesh structure of the investigated absorber.

The optical fractal absorber can be utilized in contexts where heightened light absorption holds critical significance. Potential applications encompass thermal photovoltaics, photodetectors, solar cells, and various other devices hinging on the interplay between light and matter. Crafting an optical fractal absorber within the visible and near-infrared (IR) spectrum involves the meticulous construction of a framework adept at effectively capturing light spanning those wavelengths. Figure 1 depicts the geometric configuration of the investigated fractal absorber being studied. The analyzed fractal absorber was created using a central square pillar enclosed within a cylindrical structure featuring a square hollow. This study aimed to explore the optimal parameters for the components of the investigated absorber. The primary goal was to create a fractal absorber with both perfect absorptivity and high absorptivity across an ultra-wideband spectrum. Specifically, Figure 1a provides a perspective view, Figure 1b offers a side view, Figure 1c presents a top view of the designed absorber, and Figure 1d displays the side view of the centrally positioned square pillar in the design. This endeavor hinges on the application of fractal geometry principles, a strategy renowned for augmenting the interaction between light and matters by virtue of intricate, self-replicating patterns. The general guidelines on how to design a fractal absorber are listed below:

(1) A suitable fractal geometry that exhibits self-similarity across different scales is chosen. Common fractal structures include the Sierpinski triangle, the Koch snowflake, and the Menger sponge. The choice of fractal geometry will influence the absorption properties overall. In this study, a central square pillar within a cylindrical structure with the square hollow was chosen as the fractal geometry, as Figure 1a–c show.

(2) In order to achieve a high absorptivity in the visible and near-infrared spectrum for the studied absorber, it is essential that the selected materials possess strong absorptive properties. These materials encompass metals, semiconductors, oxides, or other substances displaying robust absorption traits within the specific wavelength range of interest. The primary distinguishing feature of this study was the exclusive utilization of two materials: Ti and MgF_2_, as Figure 1a–d show.

(3) When designing a fundamental unit within the chosen fractal geometry, it is subsequently scaled down in an iterative manner to generate diverse structures and layers. Each individual layer constitutes a diminished rendition of the preceding one, consistently adhering to the fractal pattern. This process gives rise to the inherent self-similarity trait exhibited by fractals. As Figure 1c shows, the designed central square pillar was also simulated to prove that only the central square pillar was used; the absorber could not achieve the properties of both perfect absorptivity and high absorptivity across an ultra-wideband spectrum.

(4) When multiple layers and components of fractal geometry are stacked on top of each other or combined, these layers and components are constructed using the selected absorptive material. The thickness of each layer and the design of each component are adjusted according to the desired absorption wavelengths.

(5) Computational simulation tools, such as finite-difference time-domain (FDTD) or finite element methods (FEM), are employed to fine-tune the dimensions of the designed fractal structure. The objective is to attain the highest absorptivity within the specified wavelength range. Parameters such as iteration scale factor, layer thickness, and overall structure size are to be adjusted accordingly. To achieve this objective, the COMSOL Multiphysics^®^ simulation software (version 6.0), a commercially available tool, was employed to perform comprehensive numerical simulations and analyses on the structure of the absorber. Simulations were conducted to showcase the absorber’s performance and to fine-tune its design for the desired characteristics.

(6) To further amplify the absorptivity of the designed absorber, an anti-reflection coating is considered for application on the surface of the fractal structure. This coating effectively reduces light reflection, thus intensifying light capture with the absorber. However, it is important to note that the examined fractal absorber is not added on top of the investigated absorber. In order to highlight the optimized parameters within the proposed structure, the width of the central square pillar, as well as the spacing between the central square pillar and the cylindrical structure with a surrounding square hollow are to be systematically and individually adjusted while keeping the other parameters constant. This methodology was employed to pinpoint the ideal parameters for each component, ensuring that its design maximizes overall performance.

(7) If the initial design does not meet the desired absorption efficiency, the design is to be iterated by adjusting the parameters, layer thicknesses, or other geometric factors. Simulation tools can help in this iterative refinement process. At first, the absorber consisted of one Ti layer (t5 in Figure 1c) and one MgF_2_ layer (t4 in Figure 1c) arranged from bottom to left and with the optimal thicknesses of 100 nm and 30 nm, respectively. This optimization process adhered to a methodical approach in which we systematically adjusted one specific length or distance parameter at a time, as shown in Figure 1c,d, while keeping the original parameters of the other components constant. Through this iterative procedure, we evaluated the structure’s performance and desired properties as each designed parameter was varied. Figure 1e depicts the mesh structure of the investigated absorber, with the following parameters: 6465 grid nodes, a maximum grid length of 5.0 nm, a minimum grid length of 1.5 nm, a curvature factor of 0.6, 36,467 tetrahedra (finite elements), 7350 triangular elements, 708 edge finite elements, 56 endpoint elements, a minimum element quality of 0.03658, and an average element quality of 0.6117.

**Figure 2:**
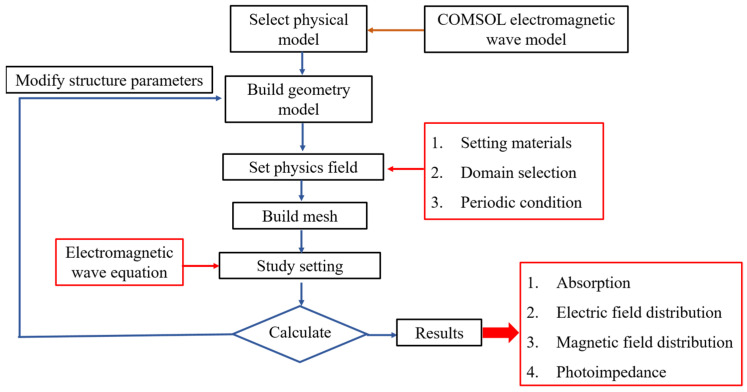
A flow diagram for the investigation of an absorber.

Figure 2 shows the schematic diagram to illustrate the step-by-step process (flow chart) for designing an ultra-wideband, perfectly absorptive fractal absorber with a central square nanopillar within a cylindrical structure featuring a square hollow. The primary focus of the analysis is to select electromagnetic waves in the frequency domain as the physical quantity for examination. Subsequently, a geometric model is constructed, material properties are defined, the solution domain is chosen, and periodic conditions are set. Finally, a mesh is created to facilitate further analysis. Regarding the geometric model, a specific layer thickness parameter is fixed, and a parameter scan is performed sequentially to determine the optimal conditions. By solving the electromagnetic wave equations, absorption, electric field distribution, magnetic field distribution, and characteristic impedance can be obtained.

## 3. Results and Discussion

**Figure 3:**
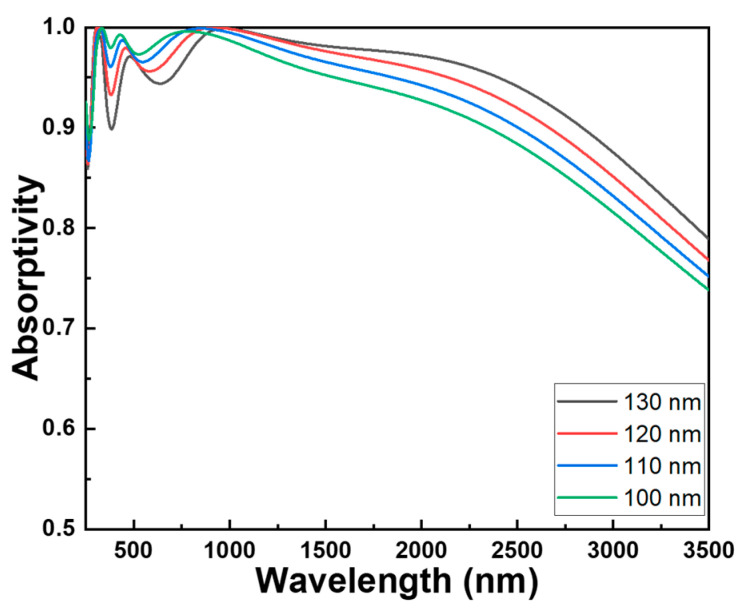
Effect of the width of the central square pillar (w1) on the absorption properties of the investigated absorber.

To verify the optimized parameters of each component material in this investigated absorber with a fractal structure, experiments for the parameter variations of each component material are individually conducted while keeping the parameters of other components constant. Figure 3, illustrating w1, the width of the central square pillar as presented in Figure 1c, can be employed to demonstrate changes in absorptivity among the various components of the absorber under investigation. This method forms the fundamental framework for determining the most suitable parameters for the absorber being studied, resulting in outstanding absorptivity and an exceptionally wide absorption spectrum. Figure 3 illustrates the alterations in the absorption spectra of the studied absorber when modifying the height of the cylindrical structure. Figure 3 also showcases the changes in absorptivity resulting from adjusting the parameter of an individual component while keeping the other parameters constant. The optimization of these parameters was conducted across a wavelength range spanning from 250 to 3500 nm. The outcomes presented in Figure 3 distinctly highlight the fact that the component parameters have apparent effects on the numerical outcomes of bandwidth and absorptivity. In order to gain a clear understanding of the absorption efficiency under various conditions, we have divided the absorption spectrum into three regions: ultraviolet (250–400 nm), visible light (400–700 nm), and infrared (700–3500 nm). Then, the maximum absorptivity within each of these regions was compared. Table 1 also presents a comparison of how the width of the central square pillar (w1) influences the maximum absorptivity of the examined absorber across various wavelength ranges. It is worth noting that, even at w1 = 120 nm, there is not a singular peak average absorptivity within the three regions. However, this particular structure exhibits the broadest bandwidth for absorptivity exceeding 90%.

The absorption spectra of the studied absorber were numerically simulated to illustrate the impact of the width of the central square pillar (w1). As the widths of the central square pillar varied at 130, 120, 110, and 100 nm, corresponding ranges where the absorptivity of the absorber exceeded 90.0% were observed within the wavelengths of 274–377 nm, 390–2838 nm, 276–2668 nm, 277–2507 nm, and 278–2347 nm. The average absorptivity is determined using the following equation [13]:

(1) $$R=\int_{\lambda_{1}}^{\lambda_{2}}\frac{R\left(\lambda \right)d\lambda }{\left(\lambda_{2}-\lambda_{1}\right)}$$

where *λ*_1_ and *λ*_2_ represent the initial and final wavelengths of the spectra where the absorptivity exceeds 90.0%. The average absorptivity within these ranges was measured at 0.965 (ranged from 274 to 2838 nm), 0.965, 0.960, and 0.959. Of particular note, even though different widths of the central square pillar were used, the graph displayed three distinct absorption peaks around wavelengths of approximately 305 nm, 456 nm, and 915 nm. The absorptivity values for these peaks were notably high at 0.999, 0.980, and 0.999, respectively. While achieving absorptivity, it exceeded 90% across the spectrum up to 2838 nm with a central square pillar width of 110 nm; the absorption rate dipped below 90% within the 377–390 nm range. This suggests that the absorber’s optimal performance is associated with these specific wavelength regions, especially when the central square pillar is 120 nm in width. Significantly, Figure 3 also illustrates that as the width of the central square pillar increases, three absorption peaks exhibit a redshift. Consequently, the detection of a redshift in these three absorption peaks indicates that their peak wavelengths transition to longer wavelengths with the augmentation of the central square pillar’s width. Propagating surface plasmon resonance refers to the excitation of surface plasmons at the interface between a dielectric material and a thin metal film, with these plasmons propagating along the metal’s surface. Therefore, the waves created in propagating surface plasmon resonance can be adjusted by varying the nanostructure geometry of the investigated absorber. This occurrence offers valuable insights into the interplay between the central square pillar’s width and the spectral response of the absorber.

Localized surface plasmon resonance is a unique plasmonic phenomenon observed when small metal nanoparticles, rather than a continuous thin film, support surface plasmon resonances. Localized surface plasmon resonance is characterized by the collective oscillations of free electrons within these nanoparticles. Therefore, changes in nanostructure dimensions, particularly at the nanometer scale, can indeed exert influence on both propagating surface plasmon resonance and localized surface plasmon resonance. Propagating surface plasmon resonance materializes when surface-bound free electrons within a metal collectively oscillate in response to incident light, thereby instigating the formation of surface plasmon waves that traverse along the interface between the metal and dielectric. Typically, this phenomenon manifests in thin metal films or on rugged metal surfaces. The interaction of incident light with propagating surface plasmon hinges upon the dimensions of the metal film and the properties of the adjacent dielectric medium. Altering the nanostructure’s dimensions can bring about shifts in the wavelength and intensity of propagating surface plasmon resonance due to variations in the effective refractive index of the surrounding medium. By contrast, localized surface plasmon resonance originates from the coordinated electron oscillations within diminutive metallic nanoparticles. This resonance manifests when the dimensions, morphology, and composition of these nanoparticles exert influence over the resonance frequency. Localized surface plasmon resonance proves to be exceptionally responsive to its immediate surroundings and can be finely adjusted by modifying the dimensions of the nanoparticles. As the nanoparticle size undergoes alterations, their localized surface plasmon resonance wavelength experiences shifts. Typically, smaller nanoparticles exhibit localized surface plasmon resonance wavelengths shifted towards the blue end of the spectrum, whereas larger nanoparticles display localized surface plasmon resonance wavelengths that shift towards the red end of the spectrum.

The observed redshift in these absorption peaks can be attributed to the interplay between propagating surface plasmon resonance and localized surface plasmon resonance. As the nanomaterial’s surface area increases, it induces a redshift in the absorption peaks of an optical absorber due to the interaction between propagating surface plasmon and incident light. This phenomenon is widely documented and is intricately linked to the coordinated behavior of electrons within the nanomaterial. When incident light interacts with nanomaterials, it triggers collective oscillations of electrons at the interface of the metal and dielectric, forming what are commonly known as surface plasmon. The plasmon resonates at specific frequencies, influenced by factors like nanomaterial size, shape, and composition. With an increase in the nanomaterial’s surface area, more electrons participate in these oscillations. Consequently, there is a rise in electron density, enhancing their involvement in the plasmon resonance. This heightened electron participation leads to a redshift in the resonance wavelength. In essence, the redshift arises from the engagement of a larger electron population in these collective oscillations. This interaction results in a reduced effective electron mass and, subsequently, a decelerated restoring force, contributing to the observed redshift in the absorption peaks.

**Figure 4:**
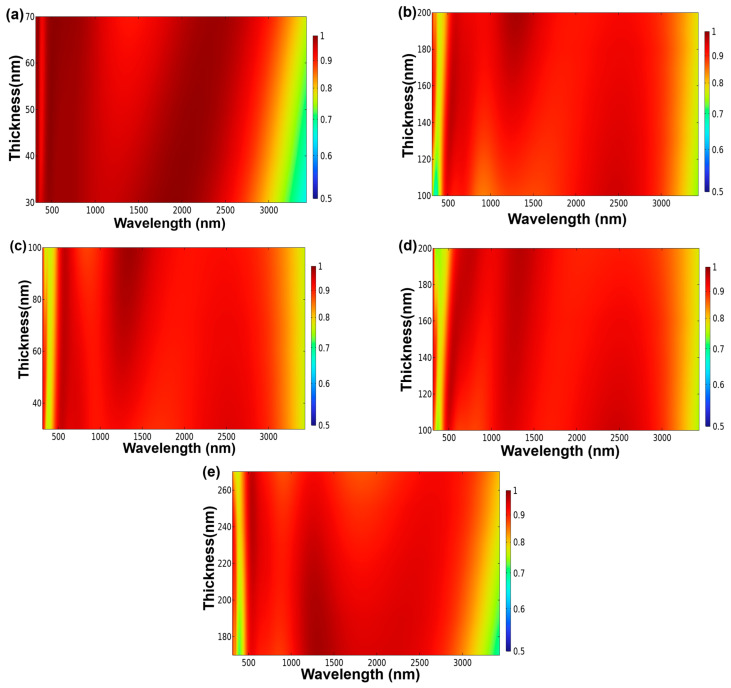
Effects of the parameter variation of each component on the absorptivity of the investigated absorber (**a**) the thickness of the lower MgF_2_ layer (t4), (**b**) the thickness of the middle Ti layer (t3), (**c**) the thickness of the upper MgF_2_ layer (t2), (**d**) the thickness of the middle Ti layer (t1), and (**e**) the height of the cylindrical Ti layer (h1).

Figure 4a–e portray the variations in absorptivity exhibited by the investigated fractal absorber. These variations arise solely from the alteration of specific parameters: the thicknesses of the lower MgF_2_ layer (t4, Figure 4a), the central Ti layer (t3, Figure 4b), the upper MgF_2_ layer (t2, Figure 4c), the outermost Ti layer (t1, Figure 4d), and the height of the cylindrical Ti layer (h1, Figure 4e). The respective parameter ranges for t4, t3, t2, and t1 encompassed values between 30 and 70 nm, 100 and 200 nm, 30 and 100 nm, and 100 and 200 nm. Additionally, the range for the height of the cylindrical Ti layer, h1, extended from 170 to 270 nm. Notably, as depicted in Figure 3, absorptivity surpasses 99% within certain wavelength ranges. This observation highlights the exceptional absorption properties of the fractal-structured absorber within these specific wavelengths. This underscores the absorber’s capacity for perfect absorption, as indicated by the absorption characteristics under investigation. To ascertain the optimal parameters for the designed fractal absorber, simulations were conducted by systematically varying individual parameters while maintaining the others constant. Figure 4a illustrates the impact on absorptivity when solely adjusting the thickness of the lower MgF_2_ layer (t4) within the range of 30 to 70 nm. This evaluation focused on the fractal absorber presented in Figure 1. As shown in Figure 4a, the enlargement of the t4 layer thickness from 30 to 70 nm resulted in diminished absorptivity within the 800–1000 nm wavelength range. The absorptivity failed to exceed 90% during this adjustment, and consequently, a thickness of 30 nm was selected for the lower MgF_2_ layer. Specifically, for a t4 value of 30 nm, the investigated fractal absorber exhibited a substantial absorptivity within the wavelength range of 276–2668 nm. 

As illustrated in Figure 4b, elevating the thickness of the central Ti layer (t3) from 100 to 200 nm yielded an expansion in the wavelength ranges below 500 nm and within the 500–1000 nm range. Additionally, a slight extension was observed in the wavelength spectrum where absorptivity exceeded 90%. Notably, when the thickness of the central Ti layer increased from 100 to 170 nm, it resulted in an augmented absorptivity within the 500–1000 nm wavelength range. Significantly, for a t3 value of 170 nm, the designed absorber exhibited substantial absorptivity spanning from 276 to 2668 nm. As a result, a thickness of 170 nm was deemed optimal for the central Ti layer. Figure 4c–e delineate the absorptivity variations observed in the investigated fractal absorber, resulting solely from adjustments in the upper MgF_2_ layer thickness (t2), the middle Ti layer thickness (t1), and the height of the cylindrical Ti layer (h1). Notably, Figure 4c,d exhibit the absorptivity outcomes corresponding to thicknesses of 50 nm and 140 nm, while Figure 4e illustrates the absorptivity performance when the height of the cylindrical Ti layer was set at 220 nm. Remarkably, it is evident that the designed absorber achieved absorptivity levels exceeding 90% within the wavelength range of 276 to 2668 nm under these specific conditions. Consequently, after careful optimization, thicknesses of 30 nm (MgF_2_) and 170 nm (Ti) were selected for layers t4 and t3, respectively. Additionally, thicknesses of 50 nm (MgF_2_) and 140 nm (Ti) were identified as the optimal values for layers t2 and t1. Furthermore, a central square pillar width of 120 nm and a cylindrical structure width of 220 nm were determined as the ideal design parameters.

**Figure 5:**
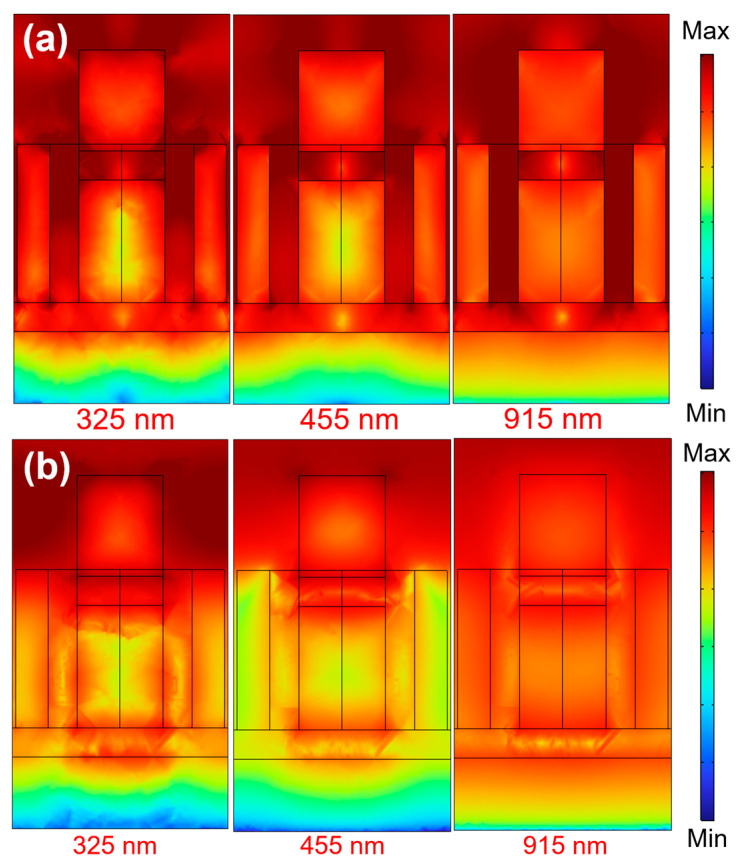
Distributions of (**a**) electric field and (**b**) magnetic field intensities under normally incident TE-polarized light at various excitation wavelengths.

In order to elucidate the fundamental physical mechanisms accountable for the notable absorptivity and remarkably wide-ranging broadband traits observed in the investigated absorber, simulations were executed to scrutinize the intensities of the electric and magnetic fields. These field intensity distributions are illustrated in Figure 5a and Figure 5b, respectively, on the xz plane. The incident light employed to excite the absorber was characterized by TE polarization and a normal direction of propagation. As depicted in Figure 3, we observed the presence of three distinct absorption peaks positioned at 325, 455, and 915 nm. Consequently, these specific wavelengths were leveraged to investigate and map the distributions of both electric and magnetic field intensities. This analysis was conducted under the illumination of normally incident TE-polarized light. These particular wavelengths were deliberately chosen since they align with the absorption peaks prominently featured in the absorption spectrum depicted in Figure 3. The presence of three peaks signifies resonance reactions taking place within the material or structure under examination. Precisely, these three absorption peaks align with distinct resonance modes at specific wavelengths. At these wavelengths, the structure or material showcases a notable enhancement in the intensity of either the electric field or the magnetic field. Within this context, the electric field within the material or structure can undergo significant intensification, leading to resonance phenomena. This heightened electric field strength holds the potential to be effectively harnessed for amplifying the interaction between light and materials. Likewise, the analysis of the magnetic field can also serve as a valuable tool for examining the magnetic field distribution at wavelengths where absorption peaks manifest. During the three specific wavelengths, the magnetic field linked with a given material or structure could undergo substantial enhancement, thereby giving rise to resonance phenomena. This heightened magnetic field strength can be strategically utilized to enhance light absorption or to manipulate the attributes of materials sensitive to magnetic fields.

**Figure 6:**
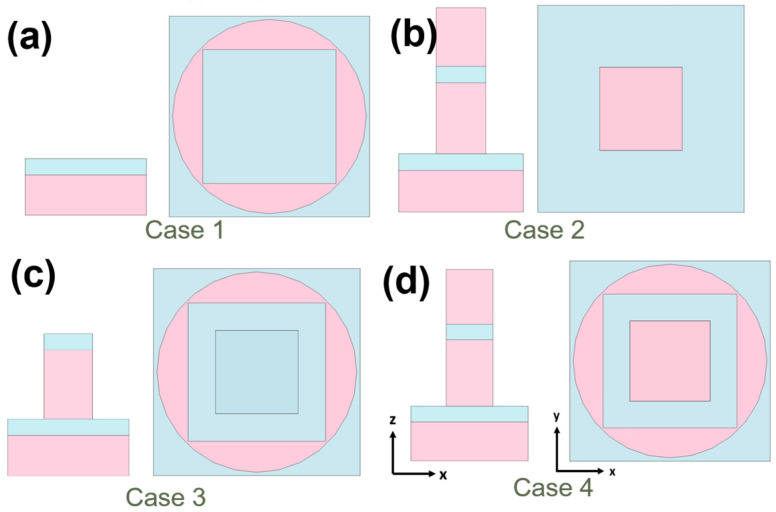
Structures used to find the relative absorption properties for different components on t5 and t4 layers: (**a**) only the cylindrical structure with a square hollow, (**b**) only the central square pillar with three layers (t1–t3), (**c**) the cylindrical structure and central square pillar with two layers (t2,t3), (**c**) top view of the designed absorber, and (**d**) the investigated structure.

The correlation between nanomaterial area and the phenomenon of propagating surface plasmon resonance is predominantly dictated by the principles of plasmonics and the interplay between light and nanoscale structures. This resonance, known as propagating surface plasmon, emerges when incident light synchronizes with the collective electron oscillations transpiring at the junction of metal and dielectric interfaces. This captivating phenomenon is profoundly responsive to alterations within the immediate surroundings. Propagating surface plasmon resonance is commonly observed in metal nanostructures or thin films. In this study, incident TE-polarized light at a normal incidence was utilized to stimulate the target absorber at wavelengths of 325, 455, and 915 nm. These particular wavelengths were chosen in reference to the absorption peaks identified in Figure 3 of the absorption spectra. Illustrated in Figure 5a, the electric field intensity distributions resulting from TE-polarized light, incident at different wavelengths and normal to the surface, demonstrate pronounced coupling effects along the surface of the examined fractal absorber. As depicted in Figure 5a, robust electric fields were evident not only on the surface of the Ti layer but also beneath the contiguous eight films. The outcomes illustrated in Figure 6 confirm that, spanning the incident light wavelengths of 325, 455, and 915 nm, the electric fields predominantly pervaded the entirety of the absorber.

Localized surface plasmon is a form of surface plasmon resonance in metallic nanostructures. When light is incident upon these metallic nanostructures, the free electrons within the metal couple with the optical field, giving rise to localized surface plasmon resonance. This resonance leads to significant absorption and scattering effects at specific frequencies for the metallic nanostructures. A distinguishing feature of localized surface plasmon resonance is that their resonant wavelengths highly depend on the shape, size, and material properties of the metallic nanostructures. Therefore, by adjusting these parameters, control over the enhancement of localized surface plasmon resonance can be achieved. Because the wavelength and strength of localized surface plasmon resonance depend on the geometry and composition of the nanostructure, and it can be tuned by adjusting the dimensions of the round nanopillars and square hollow holes. Hence, the intensity of the localized surface plasmon resonance typically escalates with an increase in film thickness until a certain threshold is reached. Beyond this point, the intensity either levels off or begins to decline. The results shown in Figure 5a confirm that, across the incident light wavelengths of 325, 455, and 915 nm, the electric fields are primarily distributed throughout the entire absorber, because of the effect of the localized surface plasmon resonance. 

Fabry–Perot cavity resonance occurs within a Fabry–Perot cavity, which comprises two parallel, highly reflective surfaces, often mirrors, facilitating multiple reflections of light between them. This resonance is utilized in optical devices for precise wavelength control and filtering. Figure 5b depicts variations in the intensities of magnetic field distributions, stemming from the Fabry–Perot cavity resonances taking place within the two layers of MgF_2_ cavities, in accordance with distinct excitation wavelengths of 325, 455, and 915 nm. The results depicted in Figure 5b distinctly indicate that the magnetic fields associated with various excitation wavelengths were distributed within the two MgF_2_ layers. Fabry–Perot cavity resonance is particularly crucial in the coupling between metallic structures and non-metallic materials. This coupling can lead to the intense localization and enhancement of light, resulting in strong absorption, scattering, and transmission effects. The Fabry–Perot cavity resonance within an absorber can be achieved by incorporating two parallel flat semi-transparent metals to act as the reflective mirrors. In the fractal absorber being investigated, the three Ti layers (t1, t3, and t5) are separated by dielectric layers made of lossless MgF_2_ layers (t2 and t4 layers). Consequently, the metal layers t1–t3 and t3–t5, separated by MgF_2_ layers, create two distinct Fabry–Perot cavities. The exciting wavelengths of 325, 455, and 915 nm satisfy the Fabry–Perot cavity resonance condition. When incident light at a normal angle interacts with the absorber, it can either exhibit destructive or constructive interferences with the light being reflected. This interaction results in distinct patterns of magnetic field distribution. These patterns clearly indicate the presence of Fabry–Perot cavity resonance within the two dielectric layers of MgF_2_. The electrical and magnetic distribution patterns shown in Figure 5a,b illustrate the Fabry–Perot cavity resonance condition by setting the excitation wavelengths at 325 nm, 455 nm, and 915 nm. 

**Figure 7:**
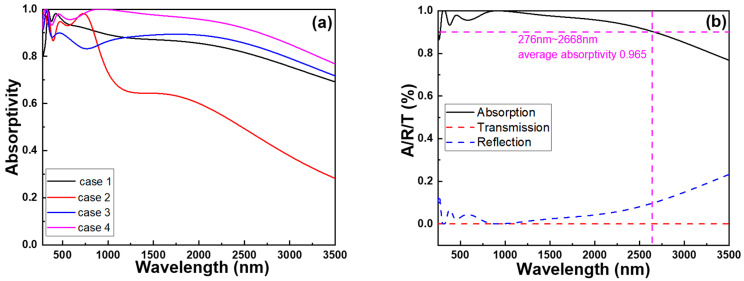
(**a**) Relative absorption spectra for the structures shown in Figure 6 and (**b**) the absorption, transmission, and reflection spectra of the investigated fractal absorber.

To ascertain the underlying cause behind the remarkable ultra-wideband and perfect absorptivity exhibited by the studied fractal absorber, various elements of the fractal structure under investigation were individually analyzed. As depicted in Figure 6, these distinct components were subjected to absorption spectrum analysis, with the simulation results illustrated in Figure 7a. In comparison to the original complete structure, Case 1′s configuration lacks the upper t1, t2, and t3 layers of the square pillar, yet retains the outer circular cylinder structure and the t4 and t5 layers. As Figure 6a shows, this modification also transforms it into a hollow structure. This alteration results in the absence of the Fabry–Perot cavity resonance, which is formed by the t1 (Ti), t2 (MgF_2_), and t3 (Ti) layers of the square pillar, associated with the square columns, leading to a reduction in absorptivity. Plasmons are coherent electron oscillations that take place within a hollow structure at specific frequencies, and these oscillations are commonly classified into symmetric and antisymmetric modes. Symmetric modes entail electrons oscillating in phase, while antisymmetric modes involve electrons oscillating out of phase. Resonance happens when the incident light frequency aligns with that of a symmetric plasmon mode. This alignment results in heightened absorptivity due to the robust interplay between plasmon oscillations and the incoming electromagnetic field. The interplay of antisymmetric modes with incident light introduces interference effects, potentially causing absorptivity fluctuations based on the system’s wavelength and geometry. 

The symmetric plasmon mode is a phenomenon that can be utilized to enhance light absorption. Its principle for enhancing absorption rate and bandwidth lies in being a type of plasmon resonance, where the free electrons within metal nanostructures undergo resonant oscillations when excited by light. The symmetric plasmon mode can generate multiple resonant modes, indicating that metal structures can resonate within different wavelength ranges. This results in an improved capacity of the component to absorb light across multiple wavelength ranges, thereby extending the absorption bandwidth of the optical absorber. Additionally, by adjusting the geometric shape, size, and arrangement of the metal structures, it is possible to control the resonant frequency of the symmetric plasmon mode. Consequently, an optimized structure can be designed according to the desired absorption range to achieve high absorption rates in the ultraviolet, visible, and near-infrared light ranges. In specific scenarios, unlike symmetric modes, antisymmetric modes might not enhance absorptivity as proficiently, because specific electromagnetic fields within the material cancel each other out. The absorption spectra in Figure 7a suggests that the antisymmetric plasmon modes in case 1 would mutually cancel out energy, resulting in a decrease in the overall absorptivity of the absorption spectrum. 

Compared to the original structure, Case 2’s configuration differs by the absence of the outer-ring cylinders, yet it retains the central square pillar intact, as Figure 6b shows. Consequently, it continues to exhibit a pronounced Fabry–Perot cavity resonance. However, due to the omission of the outer-ring cylindrical structure, it cannot mutually couple with the resonance mode of the square pillar. This leads to a substantial decrease in its absorption capacity. Compared to the original configuration, Case 3 features a modified structure with the omission of the top Ti (t1) layer on the square pillar, while still retaining the outer circular cylinder structure, as Figure 6c shows. Due to the absence of the uppermost metal layer, the square pillar in this model no longer exhibits a metal–dielectric–metal configuration. As a result, the effect of the Fabry–Perot cavity resonance becomes less pronounced, which in turn affects its absorption properties. This leads to a significant decrease in absorption compared to the absorption rate in the original setup, as Figure 7a shows. In the original configuration, the inclusion of the metal–dielectric–metal arrangement led to the formation of a resonant cavity. This cavity effectively trapped and amplified specific wavelengths, leading to increased absorption at those particular wavelengths. This phenomenon is commonly referred to as the Fabry–Perot cavity resonance. 

However, in Case 3, with the omission of the top metal layer, this resonance effect becomes less potent, causing a corresponding alteration in the absorption properties. In the original setup, as Figure 6d shows, a distinct arrangement of metal–dielectric–metal layers was positioned atop the square pillar. This arrangement seemingly contributed to the augmentation of the Fabry–Perot cavity resonance effect, ultimately resulting in an increased absorption of select wavelengths. Conversely, Case 3 involved the elimination of this uppermost metal layer (Ti, t1), thereby inducing a modification in the overall structural configuration. These findings substantiate that the analyzed fractal absorber exhibits the phenomena of propagating surface plasmon resonance, Fabry–Perot cavity resonance, and symmetric plasma occurrence within both a square pillar and a cylindrical structure with a surrounding square hollow. The presences of propagating surface plasmon resonance, Fabry–Perot cavity resonance, and symmetric plasma behavior indicates the intricate and versatile nature of this fractal absorber’s design. Each of these resonant phenomena plays a role in enhancing the absorptivity of electromagnetic waves across a broad range of wavelengths. As a result, this absorber has the potential to achieve the goal of ultra-wideband and perfect absorptivity. In Figure 7b, a comparison is presented between the absorption (A), transmission (T), and reflection ® spectra of the examined fractal absorber. The plot illustrates that the transmission ratio within the range of 250–3500 nm is zero. This absence of transmittance can be attributed to the substantial thickness of the structure, which prevents light from penetrating the lowest section of the model, thereby causing the transmittance to remain at zero. This outcome, coupled with the findings from Figure 6, suggests that the t5 (Ti) layer is capable of reflecting optical energy to enhance the absorptivity of the investigated fractal absorber.

The effect of the different structures shown in Figure 6 on the maximum average absorptivity in different wavelength ranges are also compared in Table 2. Apparently, the investigated complete fractal absorber exhibits the highest absorptivity in all three regions.

**Figure 8:**
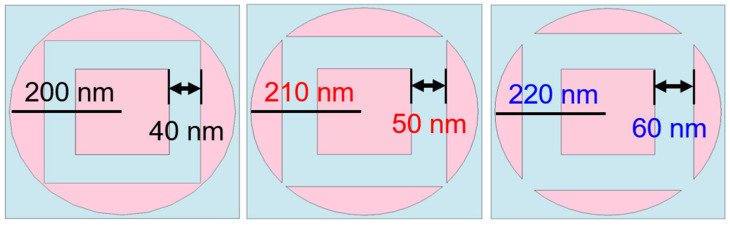
Effects of the different distances between the central square pillar and the cylindrical structure with a surrounding square hollow on the absorption properties of the investigated absorber.

**Figure 9:**
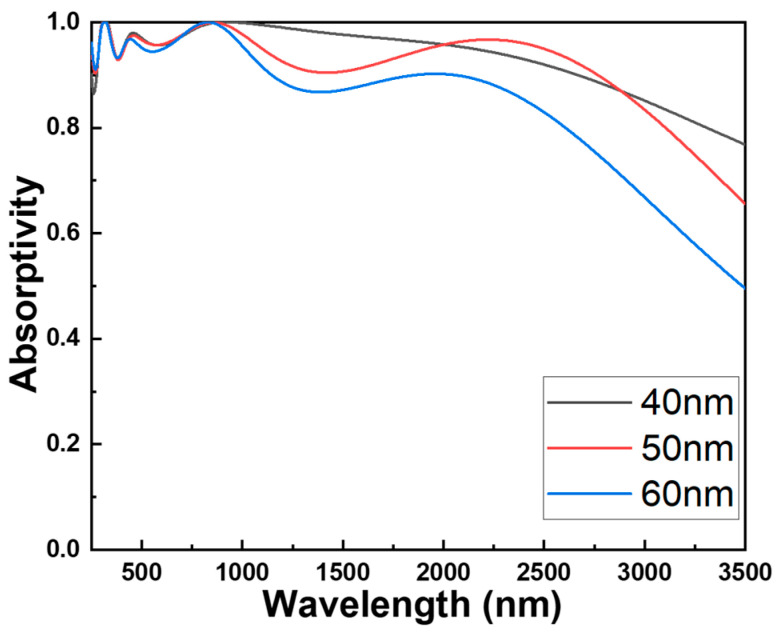
Relative absorption spectra for the structures shown in Figure 8.

To demonstrate the presence of symmetric coupling effects between the central square pillar and the cylindrical structure surrounded by square hollows, we varied their distances as illustrated in Figure 8. The comparative absorbance graphs for this fractal absorber are evident in Figure 9. An observable decrease in absorbance becomes apparent as the hollow interior of the cylindrical structure expands, transforming the outer rim into an open-loop configuration. Moreover, the degree of absorbance reduction becomes more pronounced with the gradual enlargement of the hollow square. One contributing factor is that the efficiency of the metal–dielectric–metal surface plasmon metamaterial absorber can be influenced by disruptions and reflections from external light sources. Consequently, in the open-loop configuration, these disturbances and reflections might not be effectively counteracted or compensated, leading to a diminished absorbance. Another reason is that within the open-loop structure, the external open-loop configuration cannot adequately regulate the internal coupling effects, thereby impacting absorbance performance. As the size of the gap widens, the coupling effects become less discernible. To enhance the absorption efficiency of surface plasmon metamaterial absorbers, closed-loop configurations are typically adopted. This allows for adjustments and optimizations of the metamaterial absorber based on specific application requirements. Between the central square pillar and the cylindrical structure with a surrounding square hollow, the mutual coupling of resonant modes enhances absorptivity, leading to comparatively superior absorptivity in this region compared to others.

The effects of the different distances between the central square pillar and the cylindrical structure with a surrounding square hollow shown in Figure 8 on the maximum average absorptivity of the investigated absorber in different wavelength ranges are compared in Table 3. Even at a distance of 40 nm, there is no peak average absorptivity in the 250–400 nm region. However, this structure achieves the maximum average absorptivity in the other two regions.

When aiming for high absorptivity in an absorber, the goal is to effectively capture incident light. In such scenarios, several factors come into play that result in the real part of impedance approaching unity and the imaginary part tending towards zero:

(a) Upon entering a medium, a portion of light is typically reflected at the interface due to differing refractive indices. However, for enhanced absorption, the aim is to minimize the light reflection, allowing more light to penetrate the material. An impedance value close to one (or a refractive index resembling that of the surrounding medium) helps curtail reflection.
(b) Approaching an impedance near unity aids in aligning the absorber’s impedance with that of the surrounding medium, such as air. This minimizes impedance mismatch and optimizes matching, leading to reduced reflection and the optimal coupling of light into the absorber.
(c) While an entirely imaginary impedance component signifies perfect absorption, this outcome is often impractical. Instead, minimizing the imaginary part of impedance (or absorption coefficient) indicates diminished dispersion and reduced light scattering within the material. This characteristic is vital for efficient energy absorption.

**Figure 10:**
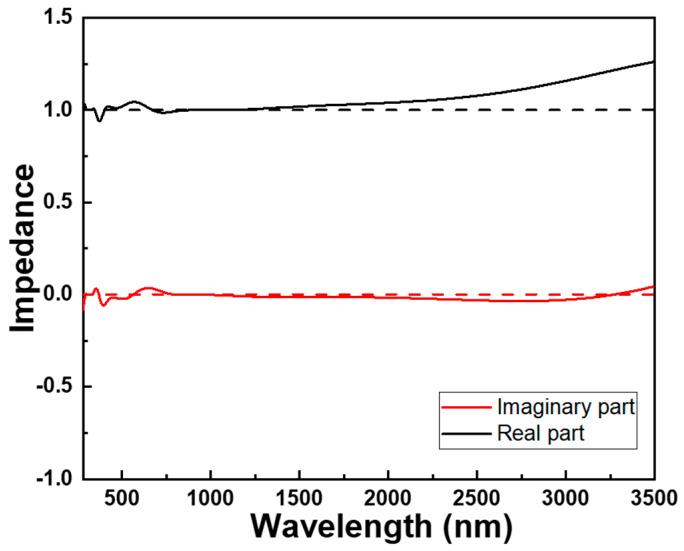
Optical impendence of the designed fractal absorber.

It is crucial to acknowledge that achieving these ideal conditions hinges on the designs and properties of an investigated absorber’s material, encompassing thickness, composition, and structure. Furthermore, the behavior might fluctuate based on the light’s wavelength and the specific attributes of the materials involved. Therefore, optical impedance is a significant parameter employed to describe the propagation characteristics of light waves within a medium and their interaction with materials. It offers insights into the energy transmission and absorption behavior of light and finds widespread applications in the field of optics. The real part of optical impedance reflects a material’s impedance to an alternating current. A real part of optical impedance close to one signifies that the material possesses very low impedance to an alternating current. In optical impedance analysis, the magnitude of the real part is contingent upon the material’s conductivity or conductivity characteristics. When a material has high conductivity, the real part of optical impedance approaches one. The imaginary part of optical impedance represents phase difference, and in this model, the imaginary part tends toward zero, indicating minimal energy loss. As illustrated in Figure 10, over the span of 250 to 2650 nm, the real component of the optical impedance gravitates towards unity, accompanied by a gradual diminishment of the imaginary component towards zero. This remarkable optical impedance profile seamlessly harmonizes with the outcomes previously observed. Notably, the extensive absorption observed across the wavelength range of 276 to 2668 nm surpassed an impressive threshold of 90.0%.

The simulation results clearly illustrate the remarkable absorption properties of the studied fractal absorber, surpassing those of many other absorbers that have been examined. This advancement can be attributed to the enhancements observed in plasmon-induced propagating surface plasmon resonance, localized surface plasmon resonance, Fabry–Perot cavity resonance, and symmetric plasma modes across the analyzed wavelength range. The angle of incident light plays a crucial role in the performance of optical absorbers, especially when dealing with thin-film or nanostructured materials. The significance of a broad incident light angle on an optical absorber arises from its capacity to amplify light entrapment, expand the range of absorbed wavelengths, diminish reflectance, and ensure performance unaffected by angle of incidence. This is due to the extended path that light follows within the absorber material when exposed to a wider incident angle. This effect becomes especially relevant in structures like thin films, where light could otherwise swiftly pass through without being fully absorbed. Distinct materials exhibit varying absorption tendencies across different light wavelengths. Enabling light to approach from diverse angles guarantees the efficient absorption of a broader spectrum of wavelengths. When light strikes the absorber material’s interface at oblique angles, the reflective properties diminish. This reduction proves advantageous when designing anti-reflective coatings or surfaces, as a greater array of angles experiences minimal reflection, thereby heightening overall absorption. The efficacy of an absorber across an extensive range of incident angles also leads to angle-independent performance. In real-world scenarios, alterations in the angle of incident light can occur due to factors such as the changing position of the sun throughout the day. 

**Figure 11:**
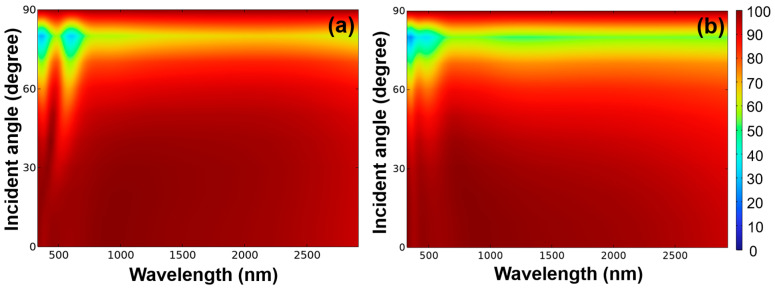
The absorptivity of (**a**) TE-polarized and (**b**) TM-polarized lights for the investigated fractal absorber with different oblique incidence angles.

An optical absorber that functions optimally, regardless of the angle of incidence, proves to be more dependable in such circumstances. The absorption performance was evaluated across incident light angles spanning from 0 to 90 degrees, taking into account both TE and TM polarizations, and the corresponding outcomes are depicted in Figure 11. When the absorber was excited under TE polarization and within the wavelength range of approximately 250 to 700 nm, the investigated fractal absorber demonstrated an absorptivity exceeding 90% over an angular range of 0 to 50 degrees. However, within the wavelength range of approximately 700 to 2700 nm, the angular range of absorptivity exceeding 90% extended to 0–72 degrees. When the absorber was excited under TM polarization and within the wavelength range of approximately 250 to 700 nm, the investigated fractal absorber exhibited an absorptivity exceeding 90% over an angular range of 0 to 53 degrees. Moreover, within the wavelength range of approximately 700 to 2700 nm, the angular range of absorptivity exceeding 90% extended to 0–63 degrees. These findings also suggest that the designed fractal absorber exhibits insensitivity to incident angles, particularly in the near-IR region. Additionally, the absorber showcases remarkable absorption performance, thanks to its highly symmetrical structure, rendering it polarization independent.

The absorption of solar radiation primarily commences in the visible light spectrum, beginning around 400 nm [15,20,21]. Additionally, achieving a 90% absorptivity over a range of incidence angles is a significant focus [15,20,21]. However, recent research results have shown that for the design of wideband absorbers, most of them only achieve an absorptivity exceeding 90% after 450 nm. Moreover, it remains challenging for most absorbers to maintain a high absorption rate beyond 2000 nm. Another issue is that these absorbers often struggle to maintain high absorption rates at incidence angles, particularly beyond 50 degrees. In comparison to these research findings, the absorber designed in this study exhibits two notable features. Firstly, it begins to demonstrate an efficiency of over 90% in the near-ultraviolet range (276 nm) and maintains this high absorption rate all the way up to 2668 nm. Secondly, within the majority of the bandwidth range designed in this study, it sustains a high absorptivity of over 90% even at incidence angles greater than 60 degrees. These exceptional characteristics are of great significance, as they open up possibilities for efficient solar energy absorption and utilization, even at challenging angles and across a broad spectrum of wavelengths. This research paves the way for improved solar energy-harvesting technologies and broader applications in renewable energy systems.

## 4. Conclusions

As the dimensions of the central square pillar were systematically adjusted to 130, 120, 110, and 100 nm, the corresponding wavelength ranges in which the absorber’s absorptivity exceeded 90.0% exhibited distinct patterns spanning from 274 to 377 nm, 390 to 2838 nm, 276 to 2668 nm, 277 to 2507 nm, and 278 to 2347 nm. The average absorptivity within these ranges was consistently measured at 0.965, 0.965, 0.960, and 0.959. The absorption spectra revealed three prominent absorption peaks. As the width of the central square pillar increased, these absorption peaks exhibited a discernible redshift, and this phenomenon serves as compelling evidence of the existences of propagating surface plasmon resonance and localized surface plasmon resonance within the structure. The electric field distribution permeates the entirety of the absorber, unequivocally substantiating the manifestation of localized surface plasmon resonance resulting from the absorber’s intentional design. Furthermore, the analyses of electric and magnetic field intensities under normally incident TE-polarized light also unveil the discernible influence of the Fabry–Perot cavity effect. However, symmetric plasma could be proven by changing the distance between the central square pillar and the cylindrical structure with the surrounding square hollow. Future research directions for fractal absorbers include expanding the wavelength range for applications in radar, communication, and sensing; exploring novel materials with enhanced absorption characteristics; creating tunable absorbers for real-time adjustable absorption properties; and integrating absorbers into diverse applications.

## Figures and Tables

**Table 1:** Maximum absorptivity in three different regions, ultraviolet (250–400 nm), visible light (400–700 nm), and infrared (700–3500 nm), as a function of the width of the central square pillar (w1).

	Wavelength (nm)	250~400	400~700	700~3500
Side Length (nm)				
100		0.963	0.983	0.900
110		0.955	0.976	0.913
120		0.949	0.965	0.927
130		0.936	0.951	0.939

**Table 2:** Maximum absorptivity in three different regions under different structures shown in Figure 6.

	Wavelength (nm)	250~400	400~700	700~3500
Different Case				
Case 1		0.917	0.946	0.828
Case 2		0.937	0.938	0.559
Case 3		0.939	0.876	0.846
Case 4		0.949	0.965	0.927

**Table 3:** Maximum absorptivity in three different regions under different distances between the central square pillar and the cylindrical structure with a surrounding square hollow in Figure 8.

	Wavelength (nm)	250~400	400~700	700~3500
Distance (nm)				
40		0.949	0.965	0.927
50		0.955	0.963	0.903
60		0.956	0.955	0.818

## Data Availability

Not applicable.
